# Jejunal Diverticulitis With Contained Perforation and Abscess Successfully Managed With Conservative Therapy: A Case Report and Review of the Literature

**DOI:** 10.7759/cureus.111181

**Published:** 2026-06-19

**Authors:** Balakrishna Ravella, Abhinay Theli, Eswar Chand Gadde, Harish Ravipati

**Affiliations:** 1 Internal Medicine, OSF HealthCare St. Mary Medical Center, Galesburg, USA; 2 Medicine, Guthrie Cortland Medical Center, Cortland, USA; 3 Internal Medicine, Ascension St. John Medical Center, Tulsa, USA; 4 Internal Medicine, Guthrie Cortland Medical Center, Cortland, USA

**Keywords:** acute abdomen, conservative management, jejunal diverticulitis, jejunal perforation, localized abscess, small bowel diverticulosis

## Abstract

Jejunal diverticulosis is a rare condition that predominantly affects elderly individuals and is often asymptomatic. However, complications such as diverticulitis, perforation, and abscess formation can result in significant morbidity. Perforated jejunal diverticulitis has traditionally been managed surgically, although increasing evidence supports conservative treatment in selected patients. We report a case of a 74-year-old female with hypothyroidism, hyperlipidemia, and Alzheimer’s dementia who presented with acute bilateral lower abdominal pain and nausea. Laboratory evaluation demonstrated leukocytosis, elevated inflammatory markers, and lactic acidosis. CT of the abdomen and pelvis revealed jejunal diverticulitis with focal perforation and an adjacent 2.4 × 3.6 cm gas-containing abscess. Given her hemodynamic stability and absence of generalized peritonitis, she was managed nonoperatively with bowel rest, IV fluids, ceftriaxone, and metronidazole. The patient improved clinically with normalization of inflammatory markers and was discharged on oral antibiotics. Follow-up imaging demonstrated complete resolution of the abscess and eventual resolution of inflammatory changes without surgical intervention. This case contributes to the growing body of evidence supporting conservative management of contained perforated jejunal diverticulitis and highlights the importance of careful patient selection and longitudinal radiographic follow-up.

## Introduction

Jejunal diverticulosis is a rare disorder characterized by acquired pseudodiverticula arising from the mesenteric border of the small intestine. The reported incidence ranges from 0.06% to 1.3% in radiologic studies and up to 4.6% in autopsy series [1-3]. Unlike Meckel’s diverticulum, which represents a true congenital diverticulum, jejunal diverticula are acquired pulsion diverticula consisting of mucosal and submucosal herniation through weakened areas of the muscular layer [4]. In simple terms, these diverticula are sac-like outpouchings of the inner layers of the bowel wall that protrude through weak points where mesenteric blood vessels penetrate the intestine.

The condition predominantly affects elderly individuals and is frequently discovered incidentally. Although most patients remain asymptomatic, approximately 10-30% develop complications, including chronic abdominal pain, malabsorption, gastrointestinal hemorrhage, obstruction, diverticulitis, abscess formation, and perforation [5-7]. Among these complications, perforated jejunal diverticulitis represents one of the most severe clinical presentations and has been associated with mortality rates approaching 20-40%, particularly in patients with delayed diagnosis, delayed treatment, diffuse peritonitis, or advanced sepsis [8-10].

Clinical diagnosis remains challenging because symptoms are often nonspecific and can mimic appendicitis, colonic diverticulitis, bowel obstruction, mesenteric ischemia, or perforated peptic ulcer disease. CT has emerged as the diagnostic modality of choice because of its ability to identify inflamed diverticula, bowel wall thickening, mesenteric inflammation, extraluminal air, and associated abscess formation [11].

Historically, perforated jejunal diverticulitis has been managed surgically. However, increasing reports in the literature suggest that selected patients with localized perforation and hemodynamic stability may be successfully treated with conservative therapy consisting of bowel rest, IV antibiotics, and close monitoring [12-14]. We present a case of perforated jejunal diverticulitis with localized abscess formation that was successfully managed without operative intervention and review the growing literature supporting nonoperative treatment in appropriately selected patients.

## Case presentation

A 74-year-old female with a medical history significant for hypothyroidism, hyperlipidemia, and Alzheimer’s dementia presented to the emergency department with sudden-onset bilateral lower quadrant abdominal pain that began on the day of presentation. The pain was sharp in nature, associated with nausea, and radiated to the upper abdomen. She denied vomiting, diarrhea, hematochezia, melena, urinary symptoms, recent travel, or abdominal trauma.

Initial vital signs revealed a temperature of 99.2°F, a heart rate of 74 beats per minute, a blood pressure of 156/62 mmHg, and an oxygen saturation of 96% on room air. Physical examination demonstrated diffuse abdominal tenderness, greatest in the bilateral lower quadrants, with voluntary guarding but without rebound tenderness or rigidity. Bowel sounds were present and normoactive. Although the patient had a history of Alzheimer’s dementia, she remained able to communicate her symptoms reliably and participate in serial abdominal examinations throughout the hospitalization.

Laboratory evaluation was notable for leukocytosis with a WBC count of 14.36 × 10³/µL, erythrocyte sedimentation rate (ESR) of 78 mm/hr, CRP of 10.3 mg/dL, and elevated lactate of 3.1 mmol/L. Hemoglobin was 14.7 g/dL, platelet count was 334 × 10³/µL, sodium was 135 mmol/L, potassium was 4.1 mmol/L, creatinine was 0.91 mg/dL, and lipase was 37 U/L. Blood cultures were obtained as part of the evaluation for possible systemic infection and sepsis, given the leukocytosis, elevated inflammatory markers, lactic acidosis, and CT evidence of perforation with abscess formation. Blood cultures remained negative throughout the hospitalization.

Contrast-enhanced CT of the abdomen and pelvis demonstrated jejunal diverticulitis with focal perforation and an adjacent 2.4 × 3.6 cm gas-containing collection, suggestive of a localized abscess (Figure 1). No evidence of diffuse pneumoperitoneum, bowel obstruction, or generalized peritonitis was identified.

**Figure 1:**
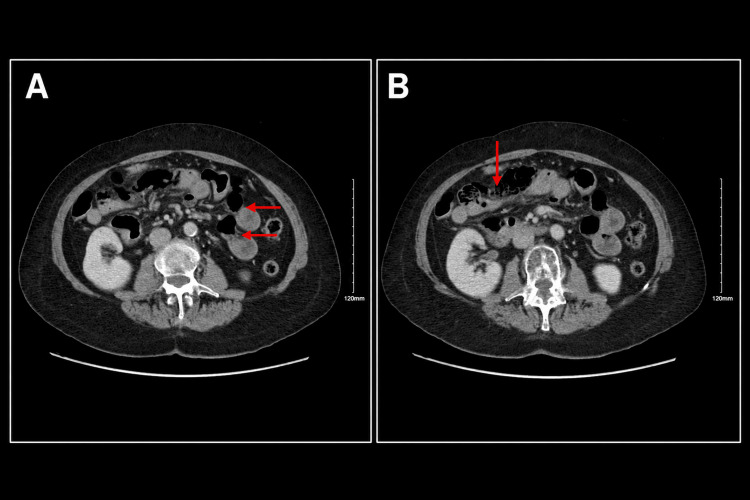
Perforated jejunal diverticulitis (A) Axial CT image of the abdomen and pelvis showing multiple jejunal diverticula, marked by red arrows. (B) Axial CT image of the abdomen and pelvis showing perforated jejunal diverticulitis with a contained abscess.

Given the CT findings, general surgery was consulted. In view of the patient’s hemodynamic stability, absence of generalized peritonitis, and radiographic evidence of a contained perforation with localized abscess formation, both the medical and surgical teams agreed that an initial nonoperative approach was appropriate, with close inpatient monitoring and a low threshold for operative intervention should clinical deterioration occur.

The patient was made nil per os and started on IV ceftriaxone 2 g every 24 hours and IV metronidazole 500 mg every eight hours. IV hydration was initiated with 0.9% normal saline at 100 mL/hour. Serial abdominal examinations and laboratory monitoring were performed, with a low threshold for operative intervention in the event of clinical deterioration.

By hospital day 2, the patient’s abdominal pain had significantly improved, and her leukocytosis had begun to resolve. Lactic acid normalized to 1.2 mmol/L, while ESR and CRP demonstrated progressive improvement. She tolerated advancement to a clear liquid diet without difficulty. On hospital day 3, she continued to demonstrate clinical improvement with near normalization of her WBC count (Table 1) and successful tolerance of a regular diet without nausea, vomiting, or worsening abdominal pain. Given her favorable clinical course, she was discharged home on oral cefdinir 300 mg twice daily and metronidazole 500 mg three times daily to complete a total 10-day antibiotic course.

**Table 1:** Laboratory value trends over the course of hospitalization showing improvement in WBC count and inflammatory markers

Parameter	Day 1	Day 2	Day 3	Reference range
WBC count (×10³/µL)	14.36	13.08	10.17	4.0-11.0
Hemoglobin (g/dL)	14.7	13.2	12.7	Male: 13.5-17.5; Female: 12.0-15.5
Platelets (×10³/µL)	334	295	266	150-450
Mean corpuscular volume (fL)	94.3	91.8	93.5	80-100
Sodium (mmol/L)	135	139	141	135-145
Potassium (mmol/L)	4.1	3.9	3.9	3.5-5.0
Creatinine (mg/dL)	0.91	0.80	0.68	0.6-1.3
Lactic acid (mmol/L)	3.1	1.2	-	0.5-2.2
Erythrocyte sedimentation rate (mm/hr)	78	60	34	Male: 0-20; Female: 0-30
CRP (mg/dL)	10.3	8.5	4.2	<0.5

Two months after discharge, follow-up CT of the abdomen and pelvis demonstrated complete resolution of the previously identified 2.4 × 3.6 cm gas-containing abscess, with no residual drainable collection, although mild residual inflammatory changes persisted in the affected jejunal segment (Figure 2A). The patient remained entirely asymptomatic. Repeat CT imaging obtained two months later demonstrated no acute inflammatory findings and revealed only a 3.7 cm diverticulum in the anterior mid-abdominal mesentery without evidence of active inflammation or perforation (Figure 2B). At the time of the most recent follow-up, the patient remained symptom-free without recurrence.

**Figure 2:**
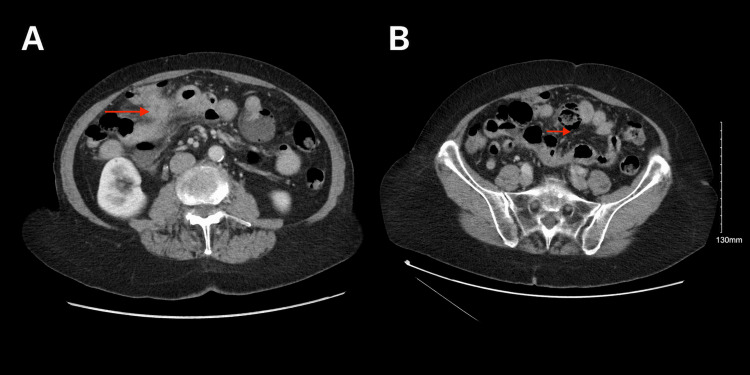
Follow-up CT images (A) Axial CT image of the abdomen and pelvis obtained two months after discharge, showing residual inflammatory changes in the jejunum but no drainable abscess. (B) Axial CT image of the abdomen and pelvis obtained four months after discharge, showing a jejunal diverticulum without any active signs of inflammation.

## Discussion

Jejunal diverticulosis is an uncommon disorder that primarily affects elderly individuals, with peak incidence occurring during the sixth and seventh decades of life [1-3]. The pathogenesis is believed to involve abnormalities in intestinal motility and increased intraluminal pressure, leading to herniation of the mucosa and submucosa through points of weakness where mesenteric vessels penetrate the bowel wall [4]. The majority of diverticula arise along the mesenteric border of the proximal jejunum [5].

Although often asymptomatic, jejunal diverticulosis may result in clinically significant complications. Diverticulitis occurs when retained enteric contents promote bacterial overgrowth and inflammation within the diverticulum. Progressive inflammation may lead to perforation, abscess formation, fistulization, hemorrhage, or bowel obstruction [6-8]. Perforation remains one of the most feared complications because of its association with peritonitis, sepsis, and increased mortality [8].

The diagnosis of perforated jejunal diverticulitis is frequently delayed because presenting symptoms are nonspecific and overlap with more common intra-abdominal pathologies. CT imaging has become the cornerstone of diagnosis and typically demonstrates focal bowel wall thickening, inflamed diverticula, mesenteric fat stranding, extraluminal air, and localized fluid collections or abscesses [11]. In the present case, CT imaging accurately identified both the inflamed jejunal diverticulum and the associated contained perforation with abscess formation, allowing prompt initiation of therapy.

Historically, perforated jejunal diverticulitis has been managed surgically, most commonly with segmental bowel resection and primary anastomosis. Surgical intervention remains indicated in patients with generalized peritonitis, diffuse contamination, hemodynamic instability, uncontrolled sepsis, or failure of medical management [8-10]. However, advances in CT imaging and improved patient selection have led to increasing recognition that certain patients with localized perforation can be managed successfully without surgery.

Several reports over the past decade have demonstrated favorable outcomes with conservative treatment in carefully selected patients. Common characteristics include hemodynamic stability, absence of generalized peritonitis, localized perforation on imaging, and close inpatient monitoring (Table 2).

**Table 2:** Published reports of conservatively managed perforated jejunal diverticulitis

Study	Age/sex	CT findings	Treatment	Outcome
Coulier et al. [11]	82/F	CT-diagnosed jejunal diverticulitis with localized perforation (microperforation) and limited extraluminal air	Antibiotics and bowel rest	Clinical and radiologic resolution
Levack et al. [12]	77/M	Localized perforation with contained extraluminal air	IV antibiotics and bowel rest	Complete recovery without surgery
Fleres et al. [13]	Case series	Localized perforations and small abscesses	Conservative management in selected patients	Successful recovery in appropriately selected patients
Kunishi et al. [14]	83/F	Localized perforated jejunal diverticulitis with peridiverticular inflammation and limited extraluminal air	Conservative management with bowel rest and IV antibiotics	Successful clinical and radiographic resolution
Present case	74/F	Jejunal diverticulitis with focal perforation and a 2.4 × 3.6 cm gas-containing abscess	IV ceftriaxone, IV metronidazole, bowel rest, and IV fluids	Resolution of symptoms and abscess without surgery

The available literature suggests that the extent of contamination and clinical stability may be more important determinants of management than the mere presence of perforation. Although no universally accepted radiologic criteria or abscess size thresholds currently exist because of the rarity of perforated jejunal diverticulitis, published reports generally support conservative management in patients who are hemodynamically stable, lack generalized peritonitis, have localized perforation, and demonstrate contained collections on imaging. Patients with localized disease and no evidence of diffuse peritonitis may be candidates for conservative therapy, whereas surgical intervention remains necessary in patients who deteriorate clinically or fail to respond to medical management [12-14].

Compared with previously reported conservatively managed cases, the present patient had a relatively large 2.4 × 3.6 cm gas-containing abscess yet remained hemodynamically stable without signs of generalized peritonitis. Similar to the patients described by Levack et al. and Kunishi et al., successful treatment was achieved with bowel rest and IV antibiotics [12,14]. However, the present report is distinguished by serial imaging demonstrating complete abscess resolution and long-term clinical recovery without recurrence. The decision not to pursue percutaneous drainage was based on the patient’s clinical stability, absence of uncontrolled sepsis, lack of diffuse peritonitis, and rapid improvement following initiation of bowel rest and IV antibiotic therapy. Furthermore, the collection was relatively small and subsequently resolved completely without invasive intervention.

The patient’s presentation closely mirrors previously reported successful nonoperative cases. Normalization of the lactic acid level from 3.1 mmol/L on presentation to 1.2 mmol/L by hospital day 2 provided objective evidence of improving tissue perfusion and resolution of the acute inflammatory process, supporting the effectiveness of conservative management. Although the erythrocyte sedimentation rate remained mildly elevated at discharge, this was interpreted in the context of overall clinical recovery, recognizing that ESR frequently normalizes more slowly than other inflammatory markers.

An additional strength of this report is the availability of long-term radiographic follow-up. Although repeat imaging at two months demonstrated residual inflammatory changes, the abscess had completely resolved, and the patient remained asymptomatic. Subsequent imaging demonstrated only a residual diverticulum without active inflammation. Few published reports provide serial imaging extending beyond the acute hospitalization, making this case a valuable contribution to the existing literature.

Collectively, the present case adds to growing evidence that carefully selected patients with contained perforation and localized abscess formation may be managed successfully without operative intervention, potentially avoiding the morbidity associated with emergency laparotomy and bowel resection.

## Conclusions

Jejunal diverticulitis with perforation is a rare but potentially life-threatening cause of acute abdominal pain in elderly patients, often presenting with nonspecific symptoms that can delay diagnosis. Early CT imaging is therefore essential for establishing the diagnosis and identifying associated complications. Although surgical intervention has traditionally been considered the standard treatment for perforated jejunal diverticulitis, growing evidence suggests that carefully selected patients with localized perforation, contained abscess formation, and hemodynamic stability may be successfully treated with conservative therapy. However, the successful nonoperative management of the 2.4 × 3.6 cm abscess described in this report should not be interpreted as evidence that all abscesses can be treated conservatively. Larger abscesses, uncontrolled sepsis, clinical deterioration, or failure of medical therapy may still warrant percutaneous drainage or surgical intervention. The present case adds to the limited but expanding literature supporting selective nonoperative management and demonstrates that conservative treatment can be a safe and effective approach when combined with appropriate antibiotic therapy and close clinical monitoring. Given the rarity of this condition, large prospective studies are unlikely; therefore, continued reporting of well-documented cases remains crucial to refining patient selection criteria and informing evidence-based management strategies.
